# Factors associated with late stage at diagnosis among Puerto Rico’s government health plan colorectal cancer patients: a cross-sectional study

**DOI:** 10.1186/s12913-016-1590-4

**Published:** 2016-08-03

**Authors:** Karen J. Ortiz-Ortiz, Ruth Ríos-Motta, Heriberto Marín-Centeno, Marcia Cruz-Correa, Ana Patricia Ortiz

**Affiliations:** 1 Department of Health Services Administration, Graduate School of Public Health, Medical Sciences Campus, University of Puerto Rico, San Juan, Puerto Rico; 2 Cancer Control and Population Sciences Program, University of Puerto Rico, Comprehensive Cancer Center, San Juan, Puerto Rico; 3 Department of Surgery, Biochemistry and Medicine, University of Puerto Rico Medical Sciences Campus, San Juan, Puerto Rico; 4 Department of Biostatistics and Epidemiology, Graduate School of Public Health, Medical Sciences Campus, University of Puerto Rico, San Juan, Puerto Rico

**Keywords:** Colorectal cancer, Stage at diagnosis, Health services accessibility, Delayed diagnosis, Primary health care

## Abstract

**Background:**

Late stage at diagnosis of cancer is considered a key predictor factor for a lower survival rate. Knowing and understanding the barriers to an early diagnosis of colorectal cancer is critical in the fight to reduce the social and economic burden caused by cancer in Puerto Rico. This study evaluates factors associated to colorectal cancer stage at diagnosis among Puerto Rico’s Government Health Plan (GHP) patients.

**Methods:**

We conducted a cross-sectional study based on a secondary data analysis using information from the Puerto Rico Central Cancer Registry (PRCCR) and the Puerto Rico Health Insurance Administration (PRHIA). Logistic regression models were used to estimate the unadjusted odds ratio (ORs) and adjusted odds ratio (AORs), and their 95 % confidence intervals (CIs). Colorectal cancer cases diagnosed between January 1, 2012 and December 31, 2012, among persons 50 to 64 years of age, participants of the GHP and with a cancer diagnosis reported to the PRCCR were included in the study.

**Results:**

There were 68 (35.79 %) colorectal cancer patients diagnosed at early stage while 122 (64.21 %) where diagnosed at late stage. In the multivariate analysis having a diagnostic delay of more than 59 days (AOR 2.94, 95 % CI: 1.32 to 6.52) and having the first visit through the emergency room (AOR 3.48, 95 % CI: 1.60 to 7.60) were strong predictors of being diagnosed with colorectal cancer at a late stage.

**Conclusions:**

These results are relevant to understand the factors that influence the outcomes of colorectal cancer patients in the GHP. Therefore, it is important to continue developing studies to understand the Government Health Plan patient’s pathways to a cancer diagnosis, in order to promote assertive decisions to improve patient outcomes.

## Background

Stage at diagnosis is considered the most important prognostic factor in colorectal cancer and is associated with a lower survival rate [1]. Results from a recent study found that colorectal cancer patients from Puerto Rico’s Government Health Plan (GHP) are diagnosed at more advanced stages, have worse survival, and had greater excess risk of death compared with Non-Government Health Plan (NGHP) patients [2]. This results justify more research to improve our knowledge and understanding of these outcomes, moreover when colorectal cancer is the leading cause of cancer death in Puerto Rico [3].

Similar to the United States, the healthcare system in Puerto Rico utilizes private healthcare providers as the principal health services providers. Most health services are funded principally through government funding (federal and local) and employer-based insurance [4]. Nearly 7 % of the island’s population is uninsured [5].

In 1994, the government of Puerto Rico started the implementation of a managed care delivery system with a GHP. The GHP’s main goal was the integration of health care for the medically indigent population to the private health sector [6, 7]. Puerto Rico’s government, through the Puerto Rico Health Insurance Administration (PRHIA), assumed the role of contracting and overseeing the private insurance companies, delegating the responsibility to provide access to health services for medically indigent persons. The GHP beneficiaries are limited to the Medicaid, Medicare eligible, and medically indigent citizens with family incomes below 200 % of the federal poverty level [8–10]. Thus, the eligibility for the GHP beneficiaries is assigned to the office of the Medicaid program within the Department of Health of Puerto Rico, based on the family income. For 2012, nearly 45 % of Puerto Rico population had the GHP of which 5 % were dual eligible (Medicaid and Medicare) [5].

It is important to highlight that GHP cancer patients have a comprehensive coverage with the special coverage provision, which seeks to facilitate the effective management of this condition. Cancer coverage under this provision begins upon the confirmation of a cancer diagnosis. Under the special coverage patients can access specialists within the network providers and to obtain treatments, therapies or testing to address their condition, without referral or pre-authorization. Nonetheless, disparities in colorectal cancer outcomes have been documented among GHP patients when compared to NGHP patients [2].

Even with the accessibility of colorectal cancer screening and the efforts to reduce the incidence and mortality of this type of cancer, the percentage of colorectal cancer screening within the recommended ages in Puerto Rico is lower than the Healthy People 2020 target of 70.5 %. According to the Behavioral Risk Factor Surveillance System during 2012, the prevalence of adults aged 50–59 and 60–64 who had ever had a sigmoidoscopy or colonoscopy was 34.1 % and 51.5 %, respectively [11]. Moreover, in Puerto Rico it has been found that having a higher level of education and having a higher income are associated with a greater likelihood of having had a screening study for colorectal cancer [12, 13]. This highlights a health inequity that justifies more research.

The Anderson-Aday model is a theoretical framework that is commonly used to understand and study health care access. This model uses a systems perspective to integrate individual, environmental, and health provider aspects associated with the decision to seek health services [14]. All these characteristics influence people to have more or less access to health services. Individual characteristics, like age and sex, and comorbidities, could represent barriers to obtain health services in a timely manner. Similarly, health system factors for example, lack of resources such as a low supply of gastroenterologists [15–18], can be a barrier to an early cancer diagnosis.

Delay in diagnosis could be related with doctor and system factors but can also be influenced by the patient’s characteristics [19]. Many studies have classified delay in cancer diagnosis and treatment into three categories: *patient delay* or *primary delay*, from first symptom to first contact with the Primary Care Provider (PCP); *doctor delay* or *secondary delay*, from the first contact with the PCP to a confirmed diagnosis; and, *system delay* or *tertiary delay*, the time from a confirmed diagnosis to the time of treatment initiation [19–23]. Doctor and system delay could give details about the health system performance, for example, characteristics of the delivery system (availability, organization, and financing). Although some studies have found that reasonable delay between diagnosis and treatment is not detrimental to patient outcomes [1, 24], other studies state that a significant delay due to deficiencies in the health care delivery system, could be an important factor in the patient’s outcomes [19, 22, 25]. Meanwhile, patient delay may be due to patients seeking health care services late and entering the health care system through the emergency room [26]. Studies have shown that patients presenting in emergency rooms tend to have shortest delays and more advanced disease [1, 26–28].

Delayed diagnosis may contribute to a later stage at colorectal cancer diagnosis and can likewise have a negative outcome on quality of life, with the use of more harmful treatments when cancer is diagnosed at an advanced stage [23, 29]. This study evaluates for the first time individuals and health system factors associated to colorectal cancer stage at diagnosis among GHP patients in Puerto Rico.

## Methods

### Data sources

The present cross-sectional study used the Puerto Rico Central Cancer Registry (PRCCR) and the PRHIA databases. The PRCCR and PRHIA have a collaboration agreement where the PRHIA provides all claims of cancer patients to the PRCCR. Colorectal cancer cases were obtained from the PRCCR, which has met the standards for completeness and data quality [3].

In situ and invasive colorectal cancer cases diagnosed between January 1, 2012 and December 31, 2012, among Puerto Rico residents aged 50 to 64 years (*n* = 548) and reported to the PRCCR were included. Colorectal cancers were defined as tumors with International Classification of Diseases for Oncology, Third Edition (ICD-O-3), codes C18.9-C18.0 for colon cancer and for rectal cancer C19.9 and C20.9. Since screening is not recommended for persons under 50 years [30], these persons were excluded. On the other hand, individuals over 64 years were not included because most of them have Medicare, therefore, a different insurance coverage. Only cases with diagnostic confirmation of colorectal cancer were included in the analysis. Patients with unknown method of confirmation, unknown stage at diagnosis and reported to the PRCCR by the death certificate only or autopsy were excluded (*n* = 35). In addition, patients who have had another cancer diagnosis before or within 2 years after the colorectal cancer diagnosis were also excluded (*n* = 12). After obtaining the eligible incident cases of colorectal cancer diagnosed in 2012, a probabilistic match using LinkPlus V.2.0 was performed to determine GHP patients and to assign all claims by patient. Once the GHP patients were identified in the PRCCR, a review was performed to classify them in order to ensure the validity of the data.

For purposes of this study, stage at diagnosis was categorized into a dichotomous category: early stage (in situ and localized) and late stage (regional and distant), using the Derived SEER Summary Stage 2000. Other patient characteristics variables considered were age at diagnosis (grouped into three categories: 50–54, 55–59, and 60–64 years), and sex. Marital status at the time of diagnosis was classified as married (including common law or domestic partner) and unmarried (never married, separated, divorced, and widowed). Primary site was categorized as colon and rectum. Comorbidity was assessed using the Charlson Comorbidity Index, which consideres 17 comorbidities found to be related with 1-year mortality, and assigned a weighted score to each comorbid condition that represents a measure of the burden of comorbid disease [31]. We used the algorithm developed by Quan et al. [32] and a Stata module to calculate the index [33]. Charlson index score was classified as 0, 1 and ≥2.

In order to evaluate health delivery system characteristics, we examined if the type of primary care had an effect in the stage at diagnosis among colorectal cancer GHP patients. Patients were classified according to the type of primary medical group (PMG) to which they belonged. Federally Qualified Health Centers (FQHC) were compared to the other PMGs. Also, GHP regions were classified according to gastroenterologist supply. The regions are composed by West, Southwest, Northern, Southeast, Metro-North, East, San Juan and, Northeast Region. Gastroenterologist’s rate per GHP region was obtained from the PRHIA and was classified as having high (≥8.00), medium (4.00–7.99), and low (0–3.99) gastroenterologist’s rate.

This study also evaluated diagnosis delay, in order to determine if patients with longer delays had more advanced stage at diagnosis. Diagnosis delay was defined as the time in days between the patient’s first contact with the health care system to a cancer diagnosis. Previous studies have also defined diagnosis delay as time from the patient’s first contact with the health care system to cancer diagnosis [1, 34]. In addition, it has been reported that diagnosis delay outcomes such as stage at diagnosis and death are not linear, thus, patients with shortest delays can have a higher risk of death [1]. Thus, we categorized the diagnosis delay variable into a categorical variable: <14 days, 14–59 days (reference), and ≥60 days.

To analyze possible patient delay we created a dichotomous variable indicating if the patient’s first contact with the health care system for a colorectal cancer diagnosis was through the emergency room. First contact with the health care system was determined as the first visit of the patient with colorectal cancer related symptoms (abdominal pain, constipation, anemia, weight loss, rectal bleeding, among others) prior to a colorectal cancer diagnosis. In the absence of this type of claims, we chose the date of the visit prior to the first gastrointestinal investigation, before the colorectal cancer diagnosis. The colorectal cancer symptoms are based on the symptoms used in another study [35]. The first gastrointestinal investigation included abdominal radiological imaging, lower gastrointestinal endoscopy and, fecal occult blood test (FOBT).

### Statistical methods

Descriptive statistics and frequency analyses were used to describe the variables of interest. To evaluate the difference in stage at diagnosis and the independent variables of interest, a Chi-square test of Fisher exact tests were used. Logistic regression models were used to estimate the unadjusted odds ratios (ORs), adjusted odds ratios (AORs), and their 95 % confidence intervals (CIs). The likelihood ratio test was used to assess the significance of interaction terms. Likewise, we assessed multicollinearity among independent variables before performing the multivariate logistic regression analysis. Statistical analyses were performed using Stata/SE version 13.1 statistical software (Stata Corp., LP., College Station, TX).

The Institutional Review Board of the University of Puerto Rico, Medical Sciences Campus, reviewed and approved this study.

## Results

In 2012, from a total of 548 cases of colorectal cancer that were diagnosed in Puerto Rico between the ages of 50–64 years, 35.06 % (190 cases) were GHP patients eligible for analyses. The median age was 59 years. There were 68 (35.79 %) cases diagnosed at early stage while 122 (64.21 %) where diagnosed at late stage. Table 1 presents the comparison of the population characteristics by stage at diagnosis. According to the bivariate analysis, there was no association between the stage at diagnosis and the variables of sex, age group, marital status and type of primary center (*p*-value > 0.05). While 46.84 % of all patients had a Charlson comorbidity index of two or more comorbidities, there was not significant difference in terms of comorbidities and stage at diagnosis.

**Table 1:** Characteristics of GHP patients, by stage at diagnosis, Puerto Rico 2012

Variable	Early stage N (%)	Late stage N (%)	Total N (%)	χ^2^ *p*-value
All	68 (35.79)	122 (64.21)	190 (100.00)	
Median age (years)^a^	59	59	59	0.7499^a^
Age group (years)				
50–54	11 (16.18)	27 (22.13)	38 (20.00)	0.475
55–59	28 (41.18)	41 (33.61	69 (36.32)	
60–64	29 (42.65)	54 (44.26)	83 (43.68)	
Sex				
Male	36 (52.94)	69 (56.56)	105 (55.26)	0.631
Female	32 (47.06)	53 (43.44)	85 (44.74)	
Marital status				
Unmarried	29 (42.65)	65 (53.28)	94 (49.47)	0.160
Married	39 (57.35)	57 (46.72)	96 (50.53)	
Charlson comorbidity index				
0	15 (22.06)	35 (28.69)	50 (26.32)	0.092
1	14 (20.59)	37 (30.33)	51 (26.84)	
≥2	39 (57.35)	50 (40.98)	89 (46.84)	
Primary site				
Colon	43 (63.24)	91 (74.59)	134 (70.53)	0.100
Rectum	25 (36.76)	31 (25.41)	56 (29.47)	
Type of primary center				
Non FQHC	60 (88.24)	109 (89.34)	169 (88.95)	0.815
FQHC	8 (11.76)	13 (10.66)	21 (11.05)	
Delay in diagnosis (days)				
<14	18 (26.47)	33 (27.05)	51 (26.84)	0.091
14–59	31 (45.59)	38 (31.15)	69 (36.32)	
≥60	19 (27.94)	51 (41.80)	70 (36.84)	
First visit at ER				
No	51 (75.00)	68 (55.74)	119 (62.63)	0.009
Yes	17 (25.00)	54 (44.26)	71 (37.37)	
Region gastroenterologist rate (per 10,000)				
High rate (≥8.00)	12 (17.65)	18 (14.40)	30 (15.54)	0.671
Medium rate (4.00–7.99)	20 (29.41)	44 (35.20)	64 (33.16)	
Low rate (0–3.99)	36 (52.94)	63 (50.40)	99 (51.30)	

^a^Wilcoxon statistics *p*-value

In regards to delay in cancer diagnosis, there were significant differences by stage at diagnosis (*p* < 0.05). Out of all patients diagnosed in early stage, 45.59 % had a diagnostic delay between 14 and 59 days, while 41.80 % of patients diagnosed at advanced stage had a diagnostic delay of more than 60 days. Similarly, when we examined the variable that indicates if the patient’s first contact with the health care system for a cancer diagnosis was or not through the emergency room, we found a statistically significant difference (*p* < 0.05) between this variable and stage at diagnosis. Among colorectal cancer patients diagnosed at late stage, 44.26 % presented at the emergency room as a first contact for a cancer diagnosis, while, only 25.00 % of patients diagnosed at early stage presented at the emergency room as a first contact for a cancer diagnosis (Table 1).

Table 2 presents the univariate and multivariate models predicting the stage at diagnosis by predictor variables. Of all factors studied, only delay in diagnosis and first visit through the emergency room were statistically significant in the univariate logistic regressions. On univariate analysis, patients who had a diagnostic delay of more than 59 days were 2.19 times more likely to be diagnosed in late stage compared to those who had a diagnostic delay between 14 and 59 days (OR 2.19, 95 % CI: 1.08 to 4.45). Whereas patients for whom the first visit was through the emergency room had 2.38 times the possibility of having a late stage at diagnosis than patients whose first visit was through a medical office visit (OR 2.38, 95 % CI: 1.24 to 4.59). Nevertheless, in the analysis that controlled for all factors considered, having a diagnostic delay of more than 59 days (OR 2.19 vs AOR 2.94) and having the first visit through emergency room (OR 2.38 vs AOR 3.48) were strong and consistent predictors of being diagnosed at late stage. Results from the multivariate logistic regression model showed that patients who had a diagnostic delay of more than 59 days had almost three-fold greater possibility of having a late stage at diagnosis than patients who had a diagnostic delay between 8 and 59 days, after adjusting for others factors (AOR 2.94, 95 % CI: 1.32 to 6.52). Likewise, patients for whom the first visit was through the emergency room had 3.48 times the possibility of having a late stage at diagnosis than patients whose first visit were through a medical office visit (AOR 3.48, 95 % CI: 1.60 to 7.60). When we evaluated primary site the univariate OR was not statistically significant (*p* > 0.05) but, in the multivariate analysis we observed that rectum cancer patients have a lower possibility of being diagnosed at late stage (AOR 0.48, 95 % CI 0.23 to 0.98) in comparison with colon cancer patients. Socio-demographic factors including age, sex, and marital status had no significant effect on being diagnosed at late stage in the study population. While, colorectal cancer patients with higher levels of comorbidity (2 or more) were more likely to have an early stage diagnosis (AOR 0.45, 95 % CI 0.19 to 1.03), but this association was not statistically significant (*p* = 0.06). In terms of variables related to the delivery of health care, like type of primary center or gastroenterologist rate, these did not reach statistical significance (*p* > 0.05).

**Table 2:** Univariate and multivariate analyses for factors associated with late stage at diagnosis, Puerto Rico 2012

Variable	Univariate OR (95 % CI)	*p*-value	Multivariate AOR (95 % CI)	*p*-value
Age group (years)				
50–54	1.00 [Reference]		1.00 [Reference]	
55–59	0.60 (0.25, 1.40)	0.234	0.59 (0.23, 1.53)	0.281
60–64	0.76 (0.33, 1.75)	0.516	0.86 (0.34, 2.2)	0.75
Sex				
Male	1.00 [Reference]		1.00 [Reference]	
Female	0.86 (0.48, 1.57)	0.631	0.92 (0.47, 1.79)	0.802
Marital status				
Unmarried	1.00 [Reference]		1.00 [Reference]	
Married	0.65 (0.36, 1.19)	0.161	0.77 (0.4, 1.49)	0.439
Charlson comorbidity index				
0	1.00 [Reference]		1.00 [Reference]	
1	1.13 (0.48, 2.68)	0.777	1.04 (0.39, 2.82)	0.933
≥2	0.55 (0.26, 1.15)	0.111	0.45 (0.19, 1.03)	0.06
Primary site				
Colon	1.00 [Reference]		1.00 [Reference]	
Rectum	0.59 (0.31, 1.11)	0.101	0.48 (0.23, 0.98)	0.045
Type of primary center				
Non FQHC	1.00 [Reference]		1.00 [Reference]	
FQHC	0.89 (0.35, 2.28)	0.815	0.7 (0.24, 2.01)	0.504
Delay in diagnosis (days)				
<14	1.50 (0.71, 3.15)	0.290	0.93 (0.39, 2.2)	0.862
14–59	1.00 [Reference]		1.00 [Reference]	
≥60	2.19 (1.08, 4.45)	0.030	2.94 (1.32, 6.52)	0.008
First visit at ER				
No	1.00 [Reference]		1.00 [Reference]	
Yes	2.38 (1.24, 4.59)	0.008	3.48 (1.6, 7.6)	0.002
Region gastroenterologist rate (per 10,000)				
High rate (≥8.00)	1.00 [Reference]		1.00 [Reference]	
Medium rate (4.00–7.99)	1.47 (0.60, 3.61)	0.41	2.05 (0.73, 5.78)	0.174
Low rate (0–3.99)	1.17 (0.50, 2.70)	0.72	1.7 (0.64, 4.49)	0.284

To assess whether there are variations between the groups in the health care delivery system variables, we used a multilevel generalized linear mixed-effects model to determine the potential effects of the health system variables (gastroenterologist rate and type of primary center) on stage at diagnosis. The multilevel models proved to have no significant effect on stage at diagnosis of colorectal cancer patients (*p* > 0.05) (data not shown).

## Discussion

Early diagnosis of cancer is a key factor to obtain the best possible health outcome. A previous study found that the majority of the GHP colorectal cancer patients are diagnosed at late stage [2]. With the purpose of understanding this finding, the main objective of this study was to assess the individuals and health system factors associated to colorectal cancer stage at diagnosis among GHP patients in Puerto Rico. This study shows that more than one third (36.84 %) of the study population had a delay in diagnosis of ≥ 60 days. The principal findings of this study was that after controlling for factors that could explain stage at diagnosis, the stronger predictor factors associated with stage at diagnosis was having 60 days or more in delay at diagnosis, and having the first contact with the health care system for a cancer diagnosis through the emergency room. These findings are consistent with other studies [27, 36–41]. These results are relevant in order to understand the factors that influence the health outcomes of this population. It has been found that there are persistent barriers related to the patients, physicians, and the health care system, that impede that patients gain access to medical care in a timely manner [15, 17]. A factor that may influence delay in diagnosis is the communication between the doctor and the patient. It has been found that the quality of a physician’s communication about cancer care could vary by patient’s social class, where the physician’s communication is worst in lower social class [15, 17, 23]. More research is needed to determine whether the communication between the doctor and the patient is indeed a factor in the delay in diagnosis for GHP colorectal cancer patients in Puerto Rico.

Another important result of this study was the high proportion of patients having the first contact with the health care system for a cancer diagnosis through the emergency room, since this increased the likelihood of being diagnosed at late stage. This result is relevant since these patients were in a group of age (50–64 years) where the use of screening tests is recommended. Furthermore, through the GHP these patients are assigned a PCP that has the responsibility to coordinate their care. This finding may be due to several factors. First, an important factor to consider is patient delay, from the first symptom to the first contact with the health care system or PCP. Patient delay is usually defined as the interval of time a patient becomes aware of symptoms before seeking medical care [23]. This finding suggests that visits to PCPs did not occurr in a timely manner. Studies have found that the socioeconomic aspects related to a colorectal cancer patient are associated to emergency presentation [40, 42–45]. Socioeconomic factors such as a low socioeconomic status, being unmarried and old age are associated to emergency presentation [40]. Studies have also found that lower socioeconomic status was related with a reduced likelihood of colorectal cancer screening [46–49]. This fact is relevant since the population of this study have a lower socioeconomic level. In a recent study we evaluated the factors associated with the use of emergency room as an entry point to the health care system among GHP colorectal cancer patients and we found that, compared to women, males had two times the possibility of an ER presentation [50]. This suggests that interventions focused on males could reduce the ER presentation.

Another possible explanation for this could be that patients did not receive physician referrals. Physician referrals are one of the most important factors for the patient to receive an early cancer detection [17]. A possible reason for a physician delay (mainly PCP’s) in providing a referral, for either a diagnostic test or a specialist assessment, may be related to the physician’s knowledge of the required colorectal cancer screening and their capacity to detect colorectal cancer early symptoms. Others barriers that could have an influence on ER presentation could include reimbursement and financial forces among PCP. In a managed care organization, the PCPs act as gatekeepers, who often have financial incentives to minimize specialty referrals [15, 17, 51], which may result in a barrier to a specialty cancer care.

Another factor that could explain, in part, that patients presenting at the emergency room had more advanced stage, is the fact that these patients have tumors that are more aggressive. This result warrants further research to understand the role and implications of the emergency room presentations in the outcomes of colorectal cancer.

We found no evidence to suggest that demographic characteristics like age, sex, and marital status were associated with stage at diagnosis. This may be due to that fact that behavior, lifestyle, or cultural context does not differ substantially among the low socioeconomic population of this study. It is worth mentioning that this population, being homogeneous in terms of health insurance coverage and similar in socioeconomic status, eliminates most of the potential confounding effects of these variables. Whereas, although not statistically significant, patients with a comorbidity index of two or more had a greater possibility to have early stage at diagnosis than patients without comorbidities. Similar findings have been found in other studies [52–54]. It is possible that these patients tend to have more medical follow-up visits, which could yield an early diagnosis [55, 56].

We also found no evidence to suggest that the health care system delivery factors evaluated (primary center and region gastroenterologist rate) were associated with stage at diagnosis. Thus, this study underscores the importance to further evaluate others variables related mainly to healthcare delivery, including organizational and structural related variables, which could have an influence on the delay of colorectal cancer diagnosis. We used the Anderson and Aday model as a framework to explain the access and utilization among GHP patients. According to the Aday and Anderson model, the characteristics of the health care delivery system consist of resources and organization elements [57]. Resource refers to the human resources and the capital allocated to health care whereas, organization means the way in which the system uses its resources. Future evaluation of other system’s characteristics that determines what happens following the entry into the system could be important in order to clarify and expand on the current findings.

Several limitations of this study must be acknowledged. First, since there are different operational definitions of delay in diagnosis, it is difficult to compare our results with other studies. Another limitation is the use of claims to estimate a patient’s first contact with the health care system, since claims data are designed for the purpose of reimbursement, not for research. However, the linkage with the PRCCR and PRHIA database, allowed us to validated claims data quality. In addition, we were able to complement demographic, clinical and tumor related variables with information obtained from the claims data, which permitted us to examine the factors associated with late stage at diagnosis in patients with colorectal cancer in the GHP. However, we were unable to include variables related to tumor aggressiveness, such as tumor grade due to a high proportion of patients with unknown tumor grade. Similarly, variables related to family history were not available. This type of variable is important since screening recommendation could vary according to the family history of cancer and should be included in subsequent studies. Finally, another limitation of the study was that we were able to analyze data from just 1 year (2012), restricting the sample size. Despite that, this was a population-based sample that included all eligible patients and diagnosis within the study period.

Various research opportunities arise from this study in order to provide further clarification and expand on current knowledge. Further studies are needed to evaluate the access to quality healthcare among GHP patients. Studying the GHP patient’s pathways to a cancer diagnosis could prove to be beneficial in gaining additional knowledge in quality healthcare access. Also, including NGHP patients could be important to evaluate the differences between GHP patients and NGHP patients.

It is important to point out that GHP provides preventive services, like colorectal screening tests without cost-sharing, but the use of colorectal cancer screening in Puerto Rico is still very low. Therefore making the screening tests accessible must be accompanied by an increased awareness. The GHP should focus attention on not only improving access to colorectal cancer screening but also changing attitudes about the importance of screening. We can conclude that providing coverage of cancer screening alone has not been sufficient to remove barriers to health care among GHP population.

## Conclusions

This study provides valuable information for policy makers to improve health outcomes among the GHP population. In 2004, Law number 230 was approved to create the University of Puerto Rico Comprehensive Cancer Center (UPRCCC), the entity in charge to execute the public policy related to cancer prevention, education, and research [58]. The UPRCCC developed a Comprehensive Cancer Control Plan to establish how to better address the cancer burden, driven by evidence-based approaches for action [59]. Developing and disseminating this type of studies help to establish specific strategies to promote screening among GHP patients, and to reduce the delay at diagnosis, among others. In addition, strengthening collaborations with entities like the Colorectal Cancer Coalition of Puerto Rico, could be an effective strategy to promote continuous programs that support colorectal cancer screening awareness.

The GHP is focused on integrated care, thus, it is expected that primary care, through a PCP, plays a central role in the health care delivery system. Consequently, primary care is a critical component of the GHP in order to provide quality access to health care services. Nevertheless, it has been observed that GHP patients are diagnosed at more advanced stages, and that the delay in diagnosis and the presentation at emergencies rooms have an influence in this outcome. It is important to raise awareness among primary physicians to facilitate patient’s access to recommended screening tests and to help GHP beneficiaries to understand the importance of performing the screening tests on time.

## Abbreviations

AORs, adjusted odds ratios; CIs, confidence intervals; FOBT, fecal occult blood test; FQHC, federally qualified health centers; GHP, government health plan; NGHP, non-government health plan; ORs, unadjusted odds ratios; PCP, primary care provider; PMG, primary medical group; PRCCR, Puerto Rico central cancer registry; PRHIA, Puerto Rico health insurance administration
